# Using DNA Barcoding to Investigate Patterns of Species Utilisation in UK Shark Products Reveals Threatened Species on Sale

**DOI:** 10.1038/s41598-018-38270-3

**Published:** 2019-01-31

**Authors:** Catherine A. D. Hobbs, Robert W. A. Potts, Matthew Bjerregaard Walsh, Jane Usher, Andrew M. Griffiths

**Affiliations:** 1 Department of Biosciences, Hatherly Laboratories, University of Exeter, Prince of Wales Road, Exeter, EX4 4PS UK; 2 ReefScan, Bristol, UK; 3 Department of Biosciences, Geoffrey Pope Building, University of Exeter, Stocker Road, Exeter, EX4 4QD UK

## Abstract

Many shark populations are in decline, primarily due to overexploitation. In response, conservation measures have been applied at differing scales, often severely restricting sales of declining species. Therefore, DNA barcoding was used to investigate sales of shark products in fishmongers and fish and chip takeaways in England. The majority of samples were identified as Spiny Dogfish (*Squalus acanthias*), which is critically endangered in the Northeast Atlantic and landings have been prohibited (although there is evidence of importation of this species). Significant differences in the species sold between retailer types were also identified, suggesting differing supply chains. The results underline issues surrounding the use of ‘umbrella’ sales terms where many species are labelled with the same designation. This denies consumer choice as purchasers cannot easily avoid declining species or those associated with high levels of toxicants. For the first time in Europe, minibarcodes are also used to identify species from dried shark fins. Despite a small sample size, analysis of UK wholesaler fins identified threatened sharks, including the endangered and CITES listed Scalloped Hammerhead (*Sphyrna lewini*). This highlights the global nature of the damaging trade in endangered shark species, in which Europe and the UK have a continuing role.

## Introduction

The elasmobranchs, which includes the sharks and rays, constitute one of the most threatened groups of vertebrates, with approximately a quarter threatened with extinction^1^. Whilst habitat loss, persecution, and climate change pose serious threats to the group, it is overexploitation that is probably the dominant driver of population declines^1^. This can be explained by the typical life-history characteristics of many species that include slow growth, long life-spans, late maturity and low fecundity, which are more typical of large growing mammals than ray-finned fish and render them especially vulnerable to overfishing. Despite this vulnerability, shark meat has been part of the human diet for thousands of years^2,3^ and is considered a traditional dish that is eaten in nations across the globe, from the eastern to western world and in both developed or developing countries. Significantly, it can also constitute an important source of protein in coastal communities, particularly those supported by small-scale artisanal fishing^4^. However, during the 1980s the worldwide shark fisheries expanded considerably, which is possibly due to the large scale economic growth in Asian economies (especially China) and resulted in an increasing demand for fins to make soup, a celebratory dish in some Asian cuisines^5^. Overall, the shark meat and fin trade, driven primarily by the need to supply the rising global demand for seafood, is predicted to accelerate if shark consumption is not regulated^6^.

According to FAO figures there has been a 42% by volume increase in global chondrichthyan meat imports between 2000 and 2011. Total world imports of shark meat stood at 121,641 tonnes volume, with a value of USD 379.8 million for the year 2011^6^, although in reality these figures are likely to be an underestimation. Inadequate reporting, artisanal market trade and the incorrect labelling of species each play a part in contributing to the discrepancies in the figures obtained^7^. The paucity of data makes it difficult to generate an accurate picture of the worldwide trade in sharks that could help inform management and conservation. The issue is compounded by the fact that the number of sharks caught from undeclared by-catch and in illegal, unreported and unregulated (IUU) fisheries probably far outweighs those from legal fisheries^8,9^.

The concern around shark population declines and extinction risk has led to 12 shark species being listed on Appendix II of the Convention on International Trade in Endangered Species of Fauna and Flora (CITES). This requires the international trade in these species to be highly regulated and includes an assessment by the exporting country that the trade will not be detrimental to wild populations^10^. These CITES listings support shark conservation at the broadest, global scale, but a wide range of regional and local regulations on shark fishing have also been applied in attempts to halt declines and promote sustainable exploitation. This includes the European Union (EU) where some fisheries are regulated by Total Allowable Catches (TACs) and for species undergoing very steep declines these can be set at zero, largely prohibiting the landings. Many vulnerable sharks have been included in this group, e.g. basking shark (*Cetorhinus maximus*), porbeagle shark (*Lamna nasus*), angel shark (*Squatina squatina*), spiny dogfish (*Squalus acanthias*) and a range of deep water sharks, in attempts to halt population declines.

Independent investigations of shark products are required to identify if prohibited species are entering the human food chain. The obstacle with such an approach is that once landed sharks are processed, the distinguishing features are removed, and typically steaks of shark are sold on to consumers. Shark is also eaten battered and fried in traditional fish and chips within the UK, and this process renders species identification difficult. Similarly, shark fins sold in Asian supermarkets and restaurants in the UK are highly processed and can be sourced as part of a complex global supply chain^11,12^. Dried fins typically originate from fisheries around the world and are exported to hubs in Asia, where they are bleached and trimmed, which again renders species identification based on morphological characteristics difficult/impossible. Therefore, we use DNA barcoding, i.e. sequencing a partial region of the cytochrome oxidase I (COI) gene, to facilitate species identification. The use of this DNA region is tied to global efforts to barcode all animal life (http://boldsystems.org/)^13^ and has found numerous applications in food authenticity, where DNA sequences from foods can be cross-referenced to databases of references^14^. The value of such approaches has been highlighted by high profile investigations that have identified mislabelling, fraud and/or uncovered the sales of endangered species^11,15–18^. However, the utility of barcoding is perhaps maximised in relation to seafood, where the global market includes trade in hundreds, if not thousands, of different species^19^.

This study aims to use COI DNA barcoding to investigate the sales of shark products within the UK to explore patterns of species utilisation and determine if endangered and/or prohibited species are being sold. In a UK context, the EU has also introduced strict legislation governing seafood labelling and the provision of key information such as commercial and scientific names to consumers, thus assuring their traceability and identification throughout the value chain (EU 1379/2013). This can also empower consumers, allowing informed choices over what species to purchase in relation to allergens, mercury (or other toxicant) levels and conservation status. However, recent work has also highlighted issues around the use of ‘umbrella’ terms to describe products. This means the use of very broad labels or designations on foods that cover a wide range of species, often with differing regulation on captures or conservation status^20–23^, and sharks remain a prime example of this problem. In the UK, EU legislation requires a designated list of commercial terms to be applied to products^24^, but terms used to describe shark products are interchangeable and cover taxonomically disparate groups. As such, consumers can often remain very uncertain about what species is being supplied and cannot make informed decisions about what product to buy. Therefore, a further aim of this study is to investigate if there is any correlation between label provided and species purchased.

## Methods

### Sample Collection & DNA Extraction

**Figure 1:**
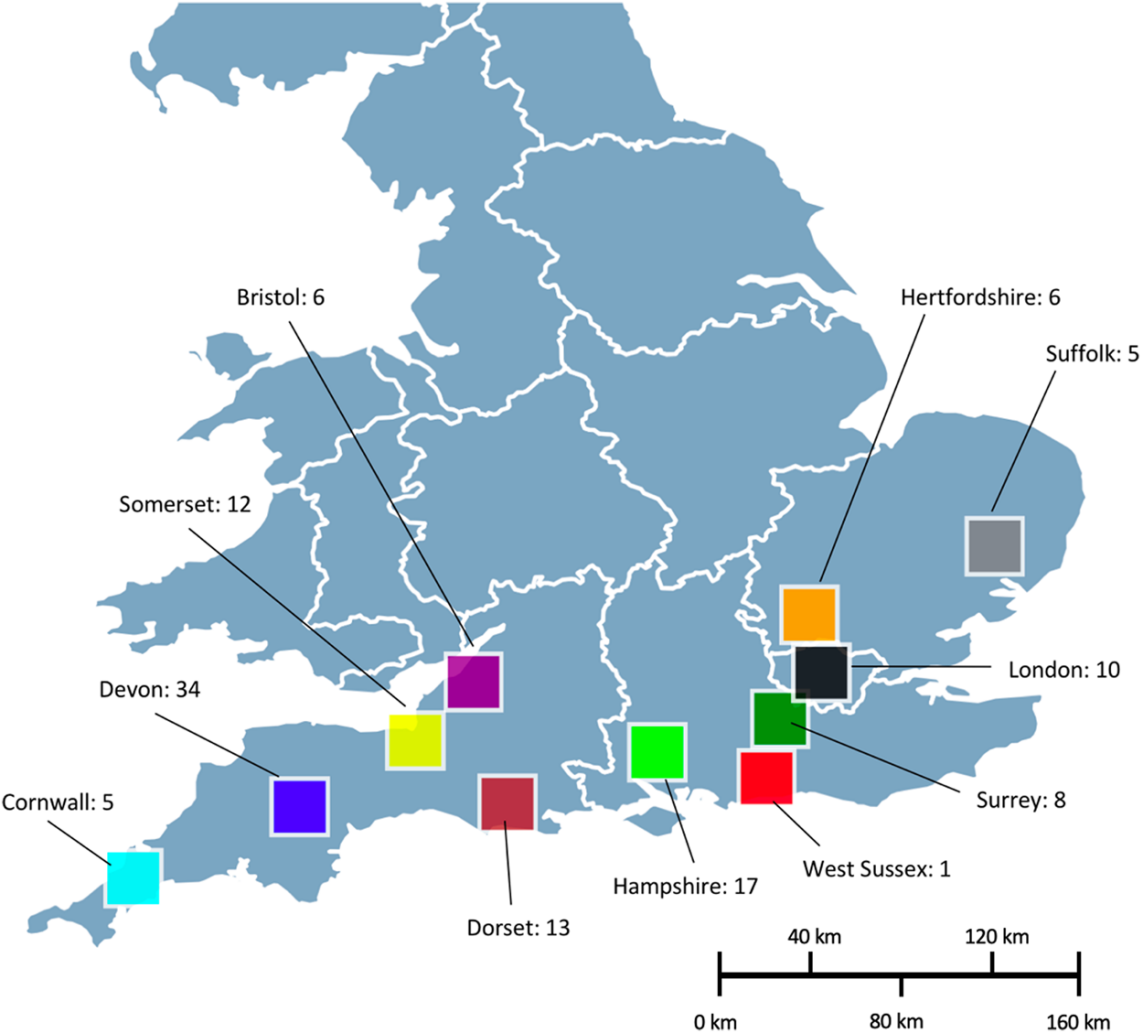
Map of Southern England summarising the sampling of shark meat products. Numbers represent sample sizes collected within each county or major city.

Between February 2016 and November 2017, a total of 117 tissue samples from shark meat products were collected from 90 different retailers (Fig. 1). These were predominately drawn from southern England as previous surveys in other regions of the UK have generally failed to demonstrate sales of these products. 78 of these were battered and fried and originated from fish and chip takeaways and 39 were either fresh or frozen and collected from fishmongers. Repeat sampling from the same retailers was kept to a low level, a maximum of two products with the same label were allowed from each retailer. In such cases a minimum of two weeks was allowed between purchases, in order to decrease the possibility of sampling the same individual/fish. Small tissue samples were dissected from each product and preserved in 2 ml labelled tubes filled with 95% ethanol and stored at −20 °C. Details of each sample were also recorded including the retailer, location, date, label/species designation and price (Supplementary Material 1).

Sampling of shark fins in the UK was more opportunistic. During the summer of 2016, 10 processed fins (that had been dried, skinned and bleached) were purchased from wholesalers in the UK, which were destined for sale in Asian restaurants or supermarkets. In March 2017, UK Customs made a seizure of dried shark fins at the UK border, originating from Mozambique, on the way to Asia. Border Force passed 30 fins for analysis (Supplementary Material 2). Samples were stored dry, at room temperature.

### Molecular Analysis & Species Identification of Shark Meat Products

Genomic DNA was extracted from samples using a chelex/proteinase K^25^ or HotSHOT^26^ protocol. In cases where this failed the DNeasy Blood and Tissue Kit (Qiagen, Manchester, UK) was also used, following the manufacturer’s guidelines. Approximately 650 bp of the COI region was amplified by polymerase chain reaction (PCR) following Serra-Pereira *et al*.^27^. One adjustment was made, M13 tailed primers^28^ were used to maximise the useful length of subsequent sequence reads; ‘F2 + T1’: (5′-TCGACTAATCATAAAGATATCGGCAC-3′) and ‘R2 + T1’: (5′-ACTTCAGGGTGACCGAAGAATCAGAA-3′)^29^. PCR products were visualized on 1% agarose gels with Safeview nucleic acid stain (NBS Biologicals) by means of ultraviolet transilluminator. Sanger Sequencing in the forward direction, utilising the tail, was then conducted to generate the DNA barcodes by GENEWIZ (Takely, UK). Sequence quality was checked using BioEdit v7.2.5^30^ and the primer regions were removed.

The resulting barcode sequences were cross-referenced using the Barcode of Life Data System (BOLD), with the species level barcode records selected (http://boldsystems.org/)^13^. Species were only assigned if the highest match was exclusive to a single species and if the degree of homology to the reference was >98% and checked by “Tree based identification”^31^. Other close matches proposed by the engine were also recorded. Subsequently, a search was also made on GenBank (http://www.ncbi.nlm.nih.gov/) with the BLASTn platform to check for the consistency of the identification. Species identified were then cross-referenced on International Union for Conservation of Nature (IUCN) Red List of Threatened Species (2018) and their conservation status was recorded.

### Molecular Analysis & Species Identification of Shark Fins

Due to the processed nature of dried shark fins, likely to result in highly degraded DNA, multiple DNA extraction approaches were utilised. Initially, a phenol-chloroform extraction, following Motteram *et al*.^32^, with an additional overnight phenol digestion after removal of the first aqueous phase, was attempted. Subsequently, a chelex/proteinase K extraction modified from Estoup *et al*.^25^, using 250 μl of chelex solution was run. When all previous attempts failed to yield DNA a Qiagen DNeasy Blood & Tissue Kit was used, with an extended overnight proteinase K digestion step. Initial testing with PCR amplification of the full length (~650 bp) COI barcode on fin extracts was unsuccessful. Therefore, a minibarcode approach (targeting ~130 bp of the gene), utilising reverse primer Shark COI-MINIR (5′-AAGATTACAAAAGCGTGGGC-3′) and following Fields *et al*.^33^, was employed. DNA barcode sequences were generated and checked as for shark meat products above.

Given the very short length of the minibarcode sequences a more conservative approach was used for identifying species in fins than meat products, which broadly follows Fields *et al*.^33^. A species level identification was only made when the highest match on both BOLD and BLAST was the same, exclusive to a single species and >99%, homology score was achieved. In cases where a species level identification could not be made most products could be assigned to genus. Where a CITES listed shark was identified the compound character attribute method was also used to confirm the identification by aligning the minibarcode against consensus sequences from Fields *et al*.^33^ and checking for the presence of the diagnostic SNPs that distinguish the CITES species from its closest relatives.

### Statistical Analysis

To compare patterns of species utilisation, the approach of Griffiths *et al*.^21^ was followed, where each retailer with products bearing the same label was used as a data point (i.e. “sample”) in the analysis. PRIMER-6^34^ was used to visualise data via non-metric multi-dimensional scaling (MDS) and calculate a non-parametric analysis of similarity (ANOSIM), as well as a similarity percentage analysis (SIMPER). All utilised the Bray-Curtis similarity measure and the program defaults (except the MDS where 1000 restarts were used). Other statistical analyses were conducted in EXCEL (2013).

## Results

### Identification of Shark Meat Products

**Figure 2:**
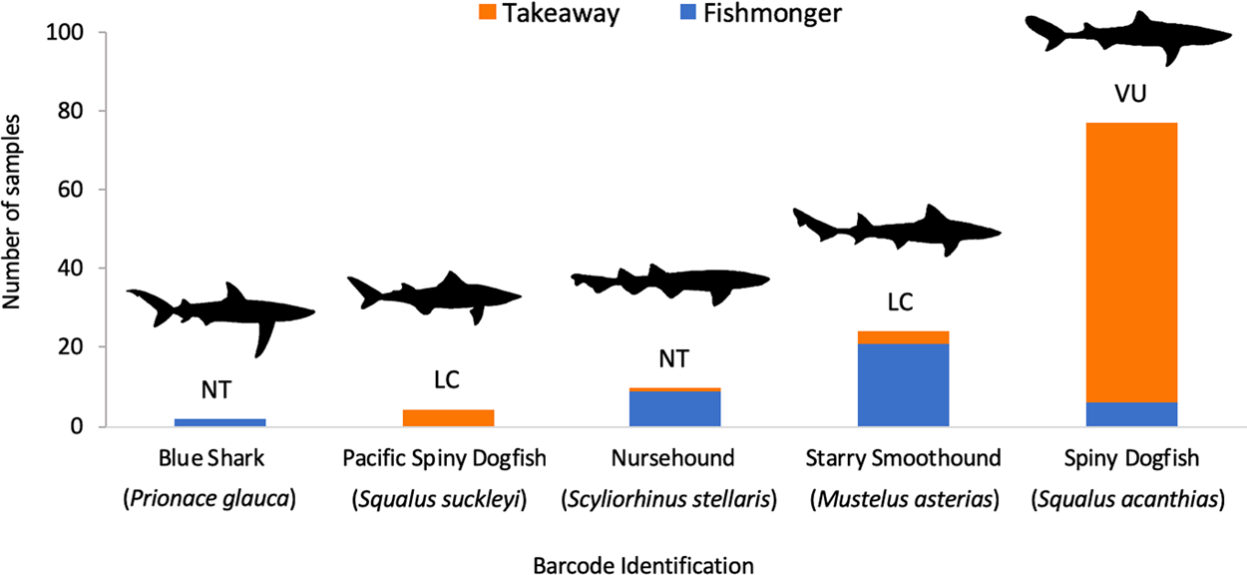
Bar chart of species identities assigned to products from takeaways and fishmongers. Bars are divided according to retail origin and the global IUCN Red List conservation status of each species is highlighted (LC: Least Concern, NT: Near Threatened and VU: Vulnerable).

Barcode sequences were generated from all 117 shark meat products (Supplementary Material 1). The average length of the edited barcodes generated was 614 bp (min: 525 bp; max: 653 bp). All produced a highest match to a single species, with >98% homology, in both the BOLD species and BLAST databases (see discussion). The meat products were identified as originating from five species: *S*. *acanthias*, *Mustelus asterias*, *Scyliorhinus stellaris*, *Squalus suckleyi* and *Prionace glauca* (Table 1 and Fig. 2). Whilst most of these species are not in threatened categories, *S*. *acanthias* that makes up the majority of samples analysed is described as globally threatened (in the IUCN Red List vulnerable category), with a decreasing population trend. Furthermore, the Northeast Atlantic sub-population is also listed as critically endangered^35^.

**Table 1:** Frequency of the shark species identified from shark meat products and shark fins.

Retailer		Blue Shark	Nursehound	Pacific Spiny Dogfish	Spiny Dogfish	Starry Smoothhound	Bull Shark	Requiem Sharks	Scalloped Hammerhead	Shortfin Mako Shark	Smalleye Hammerhead
		*Prionace glauca*	*Scyliorhinus stellaris*	*Squalus suckleyi*	*Squalus acanthias*	*Mustelus asterias*	*Carcharhinus leucas*	*Carcharhinus spp.*	*Sphyrna lewini*	*Isurus oxyrinchus*	*Sphyrna tudes*
Fishmonger	Number	2	9	0	6	21					
	Percent	5.26	23.68	0.00	15.79	55.26					
Takeaway	Number	0	1	4	71	3					
	Percent	0.00	1.27	5.06	89.87	3.80					
Wholesaler	Number						0	2	3	2	1
	Percent						0.00	25.00	37.50	25.00	12.50
Seized	Number						9	16	0	0	0
	Percent						36.00	64.00	0.00	0.00	0.00
**TOTAL**		**2**	**10**	**4**	**77**	**24**	**9**	**18**	**3**	**2**	**1**

**Figure 3:**
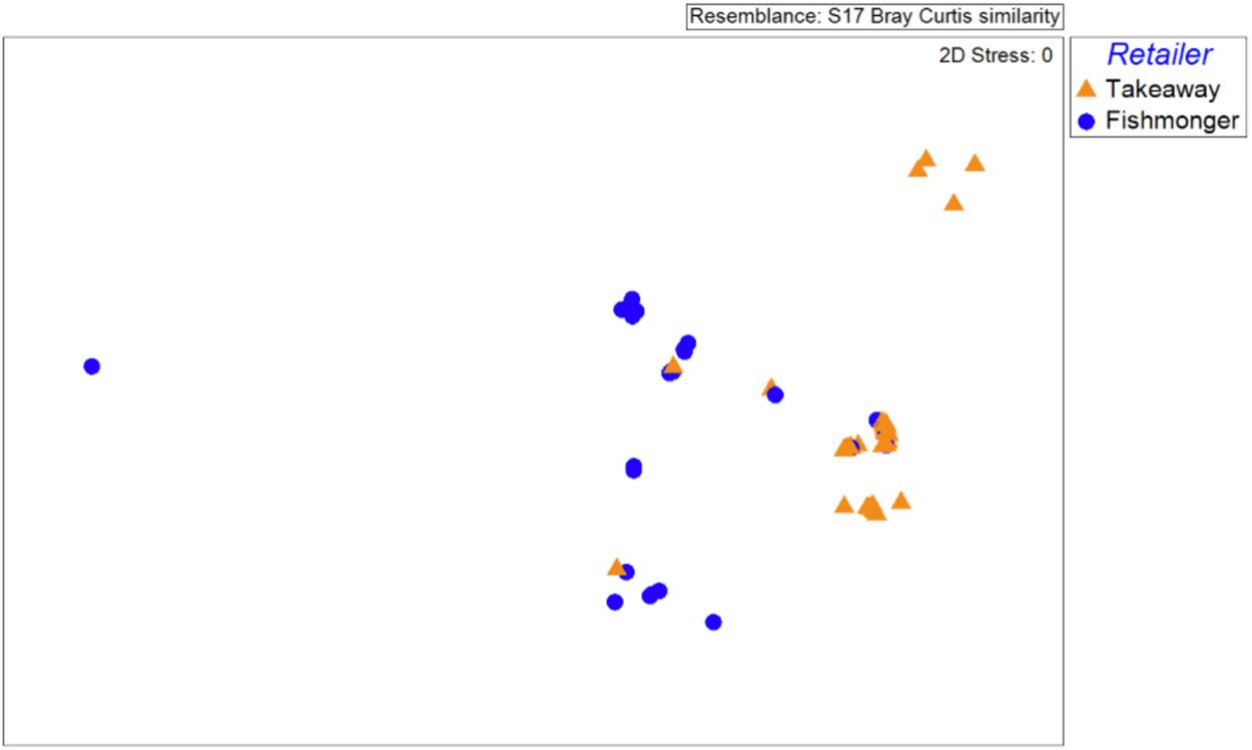
Non-metric multi-dimensional scaling plot of retailers based on species identified from shark meat products.

Comparison of species utilisation by takeaways and fishmongers by ANOSIM showed significant differences (R = 0.55, p < 0.001). This is supported by the MDS visualisation of the data (Fig. 3), where takeaways and fishmongers are generally well separated in the ordination. SIMPER analysis of retail types showed an average dissimilarity of 82.0% between takeaway and fishmonger data, with the highest contributions of dissimilarity originating from *S*. *acanthias* (43.9% contribution) *M*. *asterias*. (34.6%) and *S*. *stellaris* (12.7%). The SIMPER analysis also highlights the wider diversity of species sold by fishmongers as the within group similarity was much less for fishmongers (34.0%), compared to takeaways (71.8%). Essentially, takeaways were dominated by a single species (*S*. *acanthias* was assigned as the identity to 91.0% of products), whereas the number species sold in fishmongers was greater (although *M*. *asterias* is most common and was assigned to 51.3% of products).

### Identification of Shark Fins

**Figure 4:**
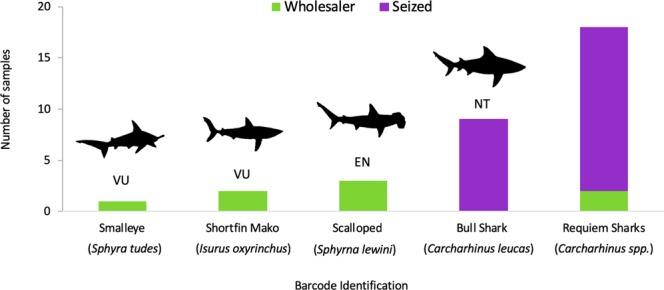
Bar chart of species identities assigned to shark fins. Bars are divided according to the source of the fins and the global IUCN Red List conservation status of each species is highlighted (NT: Near Threatened, VU: Vulnerable and EN: Endangered).

Minibarcode sequences were generated from 36 shark fins (DNA could not be extracted from four of the seized fins). The average length of the edited minibarcodes generated was 122 bp (min: 101 bp; max: 127 bp). Three minibarcodes failed to meet the more stringent requirements set in making identifications with shorter sequences (Supplementary Material 2). Many of the seized fins were assigned back to a range of closely related requiem sharks (*Carcharhinus* species) and the identification could not be made beyond genus level, as has occurred with previous applications of this minibarcode sequence^33^. However, the majority of the fins were successfully assigned back to a single species, with a consistent, highest match across both BOLD and GenBank databases. This included four species: *Carcharhinus leucas*, *Sphyrna lewini*, *Isurus oxyrinchus* and *Sphyrna tudes*, with the remaining fins assigned as *Carcharhinus species* (Fig. 4). Both the shortfin mako (*I*. *oxyrinchus*) and smalleye hammerhead (*S*. *tudes*) are listed as threatened^36^. Most significant is the discovery of the scalloped hammerhead shark (*S*. *lewini*) in the UK, as it is endangered and was effectively added to appendix II of CITES listed on 14/09/2014.

### Retail Labelling

**Figure 5:**
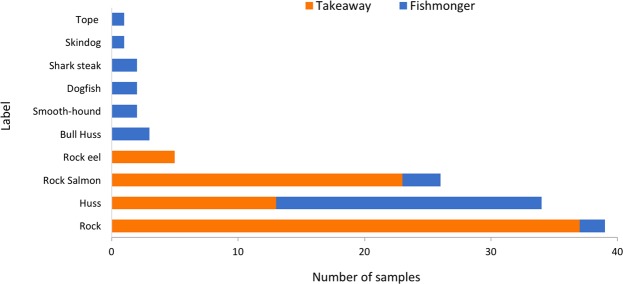
Variation in labels used by retailers (n = 115; Takeaway = 78, Fishmonger = 37, in two cases products were not labelled).

Across the sampling of takeaways and fishmongers, two products completely lacked any information, otherwise ten different labels were used, with the terms Rock, Huss and Rock Salmon, by far, the most common (Fig. 5). A Spearman’s rank correlation coefficient test was used to test for correlation between retail labels and species identity. No significant relationship was identified (r = 0.06, p = 0.92). The most frequently used label in takeaways was ‘Rock’ accounting for 47% of purchases, with the term ‘Huss’ employed for 56% of fishmonger samples. No species information was retained alongside the majority of shark fins included in this study, although three of the wholesaler fins were labelled ‘Mako’.

The official list of Commercial Designations of Fish allowed under EU legislation in United Kingdom^24^ permits the labels Dogfish, Flake, Huss, Rigg, Rock eel or Rock salmon to be used to designate all species of *Galeorhinus*, *Mustelus* and *Scyliorhinus*, and also *Galeus melastomus* and *S*. *acanthias*. This legislation (EU 1379/2013) is generally restricted to unprocessed products, so cooked samples from takeaways are not included in the following analysis. Therefore, from fishmonger samples identified as belonging to the species above, 31.4% of the products did not have an appropriate label (although many of the terms used are very similar to the commercial designations). In two cases, where official designations were not used, the labels were actually more specific: smooth-hound (and the product was identified as a *Mustelus* species of smooth-hound shark) and tope (the common name for *Galeorhinus galeus* in the UK, but this was identified as *M*. *asterias*). The remaining two products were labelled as shark steaks, and the term shark is designated for *Carcharhinus limbatus*, *C plumbeus*, *C*. *maximus*, *I*. *oxyrinchus*, *L*. *nasus*, *P*. *glauca* and *Rhincodon typus* in the UK. Both products were identified as *P*. *glauca*. So, despite widespread use of labels that do not quite correspond with official designations, there generally appears to be little mislabelling.

## Discussion

Investigating shark products in the UK has revealed a taxonomically diverse group of sharks on sale, including a number of threatened species. The most commonly identified species was *S*. *acanthias*, associated with almost all takeaway products and listed as globally threatened. Analysis of dried and processed shark fins, on route for sale in Asian restaurants and supermarkets and undertaken for the first time in the UK or Europe, revealed more threatened species, including the endangered and CITES listed scalloped hammerhead shark. Additionally, the results show that, whilst mislabelling of fresh and frozen products is rare, many retailers are not using official designations and the use of ‘umbrella’ terms is widespread, which make it difficult for consumers to make informed purchases and does not follow EU legislation regarding labelling of seafood (EU 1379/2013).

### Implications of the Analysis of Shark Meat Products

The use of COI DNA barcoding to identify species is widespread^14,37^ and, in common with such studies, species were successfully assigned to all shark meat products. The barcodes generated top matches back to single species that were identical across both BOLD and GenBank databases, but there were some difficulties unambiguously assigning species in a small number of cases. This is related to the increasing rate of species discovery^38,39^ and high levels of misidentification of sharks^39–41^. These issues have the potential to quickly render barcode records inaccurate. Two such cases appear to have occurred during this study (Supplementary Material 1). In GenBank, a record of *G*. *melastomus* appears to be misidentified and is identical to numerous *S*. *acanthias* sequences on the database (the two species belong to different orders, but grow to a similar size and have overlapping distributions). In BOLD, a record of *S*. *acanthias* originating from a food product (not whole specimen) was uploaded prior to the resurrection of S. *suckleyi*^39^ and has higher homology to sequences belonging to S. *suckleyi*. These records appear to give erroneous species identifications and were excluded in matching barcodes in this study. Such issues highlight the need for a sound biological knowledge in making barcode based identifications and the importance of ongoing curation to maintain the accuracy of baseline databases.

Significant differences in the utilisation of species were found between takeaways and fishmongers. This is suggestive of differences in the supply chain between retailers and are likely to reflect the ease of processing and shelf life, which are important factors influencing which species are supplied by different retailers, due to the highly perishable nature of seafood^42^. Fishmongers have the capacity to process a wide range of species and are less restricted by shelf life^43^. Takeaways tend to rely heavily on pre-prepared fish with long shelf life and ease of transportation, therefore preferentially buy frozen fish^44^. This is borne out in the current study as many of the fishmongers claimed to have sourced their fish locally, which is supported by the identification of these products, as all species were present, if not common, off the coast of England. A contrasting pattern emerged with takeaways, where almost all products were identified as *S*. *acanthias*, which are likely imports as the EU represents the major market for spiny dogfish meat^45^.

*Squalus acanthias* has a long history of traditional consumption in England (and other coastal regions of Northern Europe) and was once considered amongst the most abundant elasmobranchs globally, with a peak abundance in the early 20th Century^46^. Fisheries stock assessments report a decline in total biomass of >95% from baseline in the Northeast Atlantic^47^ and since the 1980’s landings in the region have declined^45^. Aggregations of females are also common (and commercially more valuable due to their larger maximum size), potentially risking unsustainable female-biased exploitation^47,48^. In response, the EU has applied a zero TAC designation since 2011, meaning landing in EU waters by EU and third country vessels is prohibited (EU 2017/127). In turn, this has driven imports from fisheries in the USA and Argentina, which initially developed to fill the gap in supply on the European market. Subsequent declines in US catch then saw development of fisheries off Canada and New Zealand. By 2001, the US exported 2700 million tonnes (92% of US dogfish reported landings) and Canada exported 1950 million tonnes (23% of Canadian dogfish reported landings) to the EU (58% of EU spiny dogfish meat imports)^49^. The trade has been supported through the continuing emergence of South American, African and Pacific suppliers^50^. It is also important to note that following exploitation, the Northwest Atlantic stock is now listed as endangered^47^ and many of these emerging fisheries have been largely unregulated^49^.

The supply of *S*. *acanthias* through an international supply chain in takeaways is also supported by observations made during sample collection: the relatively high cost of products, frequent use of frozen meat and informal discussions with some retailers who mentioned difficulties with international supplies. The identification of *S*. *suckleyi* in a small number of takeaway products, which is only found in the North Pacific, also supports the role of importation in supplying takeaways. Given the complexity and cost of importing *S*. *acanthias*, alongside the large carbon footprint it generates and the species’ threatened conservation status, it seems surprising that sales of *S*. *acanthias* remain so common, especially when fishmongers appear to sell local sharks that are not associated with these problems. These results bear some similarities to work highlighting the role of climate change in altering local catches, but where consumers continue to demand traditional seafood/species, which drives imports from areas where they are now more abundant^51,52^. Importantly, what differentiates this case is the apparent role of overfishing, not climate change, in driving declines. This is linked to the life-history of *S*. *acanthias* and aggregating habit of females that render it especially vulnerable to over-exploitation^47^.

The importation of *S*. *acanthias* from outside the Northeast Atlantic has reduced fisheries pressures on the depleted stock in the region and means the zero TAC on the species is not being broken. Although, to keep up with demand, supplies have to come from elsewhere and the species remains globally threatened. A critical question for ethical consumers then becomes: are products sourced from better managed stocks, where population trends are more positive, or other regions where exploitation is unregulated, or where stocks have threatened status? This was impossible to answer with labelling in the takeaways. It also creates a situation where demand for *S*. *acanthias* could more easily, and cheaply, be supplied by breaking the zero TAC locally in the UK/EU. Six fishmonger products were identified as *S*. *acanthias*, and should perhaps be viewed with more concern. The results in this paper already suggest more local supply chains in these retailers, therefore, it seems plausible these products have originated from the critically endangered Northeast Atlantic stock. This may not be prohibited exploitation, as in 2016 and 2017 a project promoting avoidance behaviour of *S*. *acanthias* by fishermen was run and vessels participating in the project were allowed to land very limited quantities of spiny dogfish (EU 2016/72; EU 2017/127). Unfortunately, previous investigation has generally suggested low levels of genetic divergence across much of the species range^39^ and the COI barcode does not have the resolution to identify population or region of origin in *S*. *acanthias*. However, questions surrounding stock of origin and prohibited landings could potentially be further investigated through the development of a panel of highly discriminatory single nucleotide polymorphisms under selection, with which to assign individuals back to their natal populations^53^.

### Implications of the Analysis of Shark Fins

The use of minibarcodes is becoming more prevalent, especially as interest grows in more degraded sample types (not just foods, but museum specimens, palaeontology etc.)^54^, including applications in environmental DNA and environmental surveying^55^. The fact that minibarcodes were generated for the majority of processed fins included in the study and that half of them could be assigned to the species level is positive. There are obvious methodological limitations in using such a short region of DNA to make species identifications^54^, which are aptly illustrated by difficulties distinguishing between closely related *Carcharhinus* sharks in the current study. However, it is important to consider that the primers employed were originally designed to target a variable and discriminating region of the COI gene^33^ and have been successfully employed in a number of studies, specifically to identify CITES listed species^11,12^.

The fins from Mozambique seized by UK Border Force represent one of the most significant seizures of recent years (100 kg in total) and permission was obtained to analyse a small sub-set of these. They all belong to requiem sharks (specifically the genus *Carcharhinus*) and are generally consistent with the shark fauna of the western Indian Ocean, around Eastern Africa. Arguably, it is the analysis of fins destined for sale in the UK that have produced some of the most interesting results. Despite the very small sample size, a range of threatened species were detected, most notably this includes the endangered, CITES listed scalloped hammerhead. Previous investigations of fins in Asia and North America have detected a similar range of sharks, including CITES listed species^33,56,57^. Recent, large scale investigations have shown the scalloped hammerhead to be the fourth most commonly identified shark from minibarcode analysis of over 9,000 shark trimmings collected from vendors in Hong Kong^12^. Therefore, this result is consistent with patterns of international trade and supplies of processed fins from Asia. It is interesting to speculate if the samples analysed here are the result of a prohibited international sale. The effective listing date for scalloped hammerhead in appendix II of CITES occurred on 14/09/2014, almost two years before the samples were purchased, but the storage time of dried shark fins can be years^58^. In discussion, that wholesaler revealed they did not have CITES permits and that the fins had been imported from Asia within the previous year, suggesting a prohibited import. When new CITES listings have been applied to other groups^59,60^, the levels of compliance have initially been low. Therefore, it will be informative to check how fast, and to what degree, recent CoP16 and CoP17 listings of sharks that includes the scalloped hammerhead lead to declines of trade in the future. These results represent the first analysis of processed shark fins in the UK, or Europe. Despite the small sample collection, it has demonstrated the sale of threatened sharks, highlighting the global nature of the damaging trade in endangered species. It further emphasises the need for a larger-scale investigation to properly characterise patterns of shark fin utilisation/sale in this region.

### Implementation of Labelling Legislation and the Use of “Umbrella” Sales Terms

Surveying fishmongers showed that almost a third of labels did not conform to the designations allowed in the UK (although many of the terms were close in terms of language). Despite this, all of the products were identified as one of the species included in the official list of Commercial Designations of Fish in United Kingdom^24^ and the mislabelling rate was negligible. This is similar to investigations of skate and ray products in the UK, where designations are broad and mislabelling is generally low^21^, but sharply contrasts with other investigations of elasmobranch products in the EU, including the case of “palombo” in Italy. Palombo is designated only for *Mustelus mustelus* and *M*. *asterias* and was associated with very high level of mislabelling (78%)^15^. This underlines how it is not just the presence of labels that is important: specificity is key, as it determines exactly how informative a label is to consumers, which in turn can strongly influence the level of mislabelling. However, the low level of mislabelling identified in the current study does not reveal the whole story, as current labelling legislation in EU establishes the obligation to indicate not only the commercial name, but also the species (i.e. scientific name) of fresh, frozen, smoked and dried seafood products (EU1379/2013). Species information was completely absent in all fishmonger samples. This creates a situation where the commercial designations become an ‘umbrella’ for many species, as was demonstrated here, where taxonomically disparate groups of sharks are sold under the same designation.

The UK situation is even more confusing for consumers as six different labels can designate a large number of sharks (species of *Galeorhinus*, *Mustelus* and *Scyliorhinus*, and also *G*. *melastomus* and *S*. *acanthias*). This is one of the broadest/least informative labels on the official list of Commercial Designations in the UK^24^. The term ‘shark’ is also used to designate a different group of larger growing species, including *L*. *nasus*, *C*. *maximus* and *R*. *typus*, for which zero TAC and CITES listings are in place. Attempts to establish if there was any correspondence between the label used and the species purchased, that may help make more sense of designations, found no significant relationship. The use of such ambiguous labels makes it extremely difficult for consumers to exercise a right to avoid species of higher conservation concern or those associated with specific health issues. Seafoods have been associated with a range of different allergens^61^ and there has been increasing concern about levels of toxicants in fish, from emerging worries relating to microplastics^62^ to long-standing concerns about heavy metals levels^63^, which have been shown to vary according to species^64^. Indeed, mercury appears to bioaccumulate in some sharks, reaching levels that exceed governmental safety limits for human consumption^65^, a finding that has been replicated in some large growing species of shark in Europe^66^. The use of such ambiguous, ‘umbrella’ labels goes contrary to the spirit of recent EU legislation that has sought to empower consumers and protect them from mislabelling and fraud.

## Conclusions

Investigation of diverse shark products and retailers in the UK has revealed differing patterns of species utilisation, which are suggestive of different supply chains. Fishmongers are generally associated with the widest diversity of products, which are consistent with the UK shark fauna and support the idea of more local supply chains. Conversely, fish and chip takeaways are dominated by sales of *S*. *acanthias*, a species of significant conservation concern. It is largely governed by a zero TAC and restrictions on landings in the EU, suggesting more complex, international supply chains. Whilst little mislabelling was identified among fresh and frozen products, full compliance with EU labelling legislation was not observed. In particular, these shark products in the UK are covered by relatively complex label designations, leading to ‘umbrella’ terms that include sharks from taxonomically disparate groups. This denies consumers the right to avoid species of higher conservation concern or those associated with health or food quality issues. Analysis of a small set of dried and processed fins from a UK wholesaler, part of the supply chain to Asian restaurants or supermarkets, revealed a completely different set of species. Many of these are not found in UK waters and are of threatened and endangered status, emphasising the need for further investigation of sales of prohibited sharks of high conservation concern within Europe. Overall, this study highlights the role that the UK plays in the sale of threatened sharks and how ‘umbrella’ designations on products can frustrate consumer choice.

## Supplementary information

Supplementary Information 1

Supplementary Information 2

## Data Availability

All data generated or analysed during this study are included in this published article (and its Supplementary Information files).
